# Kontinuitätserhalt des Nervus cochlearis bei der retrosigmoidalen ablativen Osteotomie des inneren Gehörgangs bei fortgeschrittenen Vestibularisschwannomen

**DOI:** 10.1007/s00106-021-01116-y

**Published:** 2021-11-23

**Authors:** Katharina Schaumann, A. Albrecht, B. Turowski, C. Hoffmann, J. F. Cornelius, J. Schipper

**Affiliations:** 1 Universitätsklinik für Hals‑, Nasen- und Ohrenheilkunde und Poliklinik, Heinrich-Heine-Universität Düsseldorf, Moorenstraße 5, 40255 Düsseldorf, Deutschland; 2 Institut für diagnostische und interventionelle Radiologie, Heinrich-Heine-Universität Düsseldorf, Düsseldorf, Deutschland; 3 Universitätsklinik für Neurochirurgie, Heinrich-Heine-Universität Düsseldorf, Düsseldorf, Deutschland

**Keywords:** Akustikusneurinom, Cochleaimplantat, Koos-Klassifikation, Hörrehabilitation, BERA (Brainstem Evoked Response Audiometry), Acoustic neuroma, Cochlear implant, Koos classification, Auditory rehabilitation, BERA (Brainstem evoked response audiometry)

## Abstract

Ausgewertet wurden 86 Patienten mit einem retrosigmoidal mikrochirurgisch resezierten Vestibularisschwannom im Tumorstadium Koos II–IV. Es zeigte sich, dass im Bereich des inneren Gehörgangs in über 2/3 der Fälle der Nervus cochlearis dem elektroneurographisch leicht zu identifizierenden Nervus facialis in immer wiederkehrenden ähnlichen Verlaufsmustern folgte. Ausgehend vom Fundus erleichterte dies die frühzeitige Identifizierung und damit den Kontinuitätserhalt des Nervus cochlearis im Verlauf des inneren Gehörgangs. Dies war vor allem dann von Bedeutung, wenn ein sicherer Funktionserhalt aufgrund der Tumorgröße oder -formation trotz intraoperativer Ableitung somatosensorischer Potenziale nicht sicher gewährleistet werden konnte, aber die Möglichkeit einer späteren Hörrehabilitation mit einem Cochleaimplantat bestehen bleiben sollte. Präoperative Magnetresonanztomographie(MRT)-Sequenzen ergaben zwar in einigen Fällen einen Hinweis auf die möglichen Nervenverläufe, die intraoperative Darstellung im inneren Gehörgang war der MRT aber überlegen.

## Abstract (en)

The data of 86 patients with retrosigmoid microsurgical resection of vestibular schwannoma in tumor stage Koos II–IV were evaluated. In more than two thirds of the cases it was shown that the cochlear nerve followed the facial nerve, which is easily identified by electroneurography, in recurrent similar patterns in the region of the internal auditory canal. Starting from the fundus, this facilitated early identification and thus preservation of continuity of the cochlear nerve in the course of the internal auditory canal. This was of particular importance when safe functional preservation could not be guaranteed due to tumor size or formation despite intraoperative derivation of somatosenoric potentials, but when the possibility of subsequent hearing rehabilitation with a cochlear implant should be granted. Preoperative MRI sequences gave an indication of the possible nerve courses in some cases, but intraoperative imaging in the internal auditory canal was superior to MRI.

Vestibularisschwannome (VS) mit Kontakt oder Verdrängung des Hirnstamms lassen sich für eine sichere Kontrolle der unmittelbar anliegenden vaskulären Strukturen chirurgisch über einen retrosigmoidalen Zugangsweg resezieren [10]. Ein interdisziplinäres Vorgehen mit Bündelung der Kompetenzen gemeinsam aus Neurochirurgie (NC) und Hals-Nasen-Ohren-Heilkunde, Kopf- und Halschirurgie (HNOKHC) hat sich aus unserer Sicht für eine Verbesserung der Ergebnisqualität bewährt [31]. Der morphologisch-anatomische Kontinuitätserhalt des Nervus facialis (Nf) und des Nervus cochlearis (Nc) garantiert zwar noch keinen entsprechenden neurophysiologischen Funktionserhalt, ist aber die Grundvoraussetzung dafür. Der Kontinuitätserhalt des Nc ist auch Grundvoraussetzung für eine mögliche spätere Hörrehabilitation mit einem Cochleaimplantat (CI), auch wenn der Patient bereits tumorbedingt einseitig hochgradig schwerhörig oder ertaubt sein sollte [36]. Dies ist gerade in den fortgeschrittenen Tumorstadien des VS (Koos III und IV) zum Zeitpunkt der Operation häufig bereits eingetreten [12]. In diesen Fällen wäre der Nc intraoperativ elektrophysiologisch nicht mehr sicher zu identifizieren, da die Welle V in der klassischen BERA („brainstem evoked response audiometry“) bzw. ABR („auditory brainstem response“) bereits präoperativ nicht mehr ableitbar ist. Somit wäre eine Stimulation des Nervs nur noch elektrisch über eine transtympanale Stimulationselektrode im Sinne von elektrisch evozierten akustischen Hirnstammpotenzialen (E-BERA) möglich. Analog zur klassischen intraoperativen BERA als Fernfeldmessung hat diese Methode allerdings den Nachteil, dass ein Antwortsignal auf die Stimulation erst nach mehreren Minuten abzuleiten ist. Zudem ist beim retrosigmoidalen Zugangsweg die Cochlea dem Operateur nicht zugänglich, sodass hierfür ein 2‑Höhlen-Eingriff nötig wäre. Um dem Operateur intraoperativ eine unmittelbarere Rückmeldung über den Funktionszustand des Nervs zu ermöglichen, hat sich gerade für den retrosigmoidalen Zugangsweg in den letzten Jahren die Ableitung von Aktionspotenzialen des Nervus cochlearis mittels Kugelelektrode direkt am Nerv als sog. Nahfeldmessung etabliert [28]. Hierbei wird mit der intraoperativ auf den Nc zu positionierenden Kugelableitelektrode die Welle 2 und 3 als Antwortpotenzial vom zweiten und dritten Neuron der Hörbahn arithmetisch gemittelt (CNAP, „cochlear nerve action potential“) [29]. Der Vorteil dieser Methode ist, dass man innerhalb weniger Sekunden ein Antwortsignal auf die akustische Stimulation bekommt [18] und als Operateur die Gelegenheit hat, durch Unterbrechung der Manipulationsprozedur, Spülung oder Dekompression deutlich zeitiger gegenzusteuern [22]. Da hierbei zur Stimulation ebenfalls ein akustischer Reiz benötigt wird, ist allerdings auch bei dieser Methode eine elektrophysiologische Identifikation des Nc bei präoperativ bereits ertaubten Patienten nicht sicher möglich, sodass gerade in diesen Fällen die Kenntnis über den intraoperativ zu erwartenden Verlauf des Nf und Nc von Vorteil wäre.

Der Nf lässt sich intraoperativ als motorischer Nerv durch elektrische Stimulation im Gegensatz zum Nc als sensorischem Nerv leicht identifizieren. Beim translabyrinthären Zugangsweg mit einer Teilablation des Labyrinths und Freipräparation des Fundus wird der Nc durch Darstellung an seinem Austrittspunkt aus der Cochlea sicher identifiziert. Der Verlauf beider Nerven (Nf und Nc) zwischen dem Austritt aus dem Hirnstamm und dem Eintritt in das Labyrinth erfolgt dabei nach bestimmten Verlaufsmustern, die es für eine sichere Identifizierung beider Nerven frühzeitig zu erkennen gilt [11]. Eine Domäne der HNOKHC beim retrosigmoidalen Zugangsweg ist dabei die endokranielle chirurgische Exploration des inneren Gehörgangs („internal acoustic canal“, IAC) über eine ablative Osteotomie. Die Kenntnis der bei jedem Patienten individuell räumlichen Projektion des knöchern eingebetteten hinteren Bogengangs („posterior semicircular canal“, PSC) zum IAC sowie der durch das intrameatale Tumorwachstum veränderte, teilweise prolongierte und lokal verlagerte Verlauf der 4 Nervenstrukturen des Nc, Nervus vestibularis inferior (Nvi) und superior (Nvs) sowie des Nf sind für den Funktionserhalt des Hörens und der Gesichtsmuskulatur entscheidend [3, 5, 20, 23, 27]. Eine Analyse der präoperativen hochauflösenden Computertomographie („high resolution computed tomography“, HRCT) des Felsenbeins (0,4 bis 0,6 mm Schichtdicke) sowie die der 3‑D-CISS-Sequenzen in der T2-Gewichtung in der MRT jeweils in 0,5- oder 0,7-mm-Schichtung sollen dazu erste Hinweise geben. In der Koos-Klassifikation wird für Tumoren der Koos-Klassifikation II die Lage der intrameatalen Nervenstrukturen zum Tumor bereits entsprechend beschrieben (Koos-Subtyp 1 A–C und Typ 2 A, B) [14].

Ziel unserer Studie ist es, analog hierzu tumorstadienübergreifend wiederkehrende Verlaufsmuster zu identifizieren und systematisch zu skizzieren. Durch die hierdurch gewonnenen Erkenntnisse über den zu erwartenden Verlauf des Nc kann dieser mit größerer Sicherheit identifiziert und erhalten werden, sodass eine spätere Rehabilitation mit einem Cochleaimplantat möglich wird. Zudem gilt es die Hypothese zu prüfen, dass für eine sichere Identifikation des Nc beim retrosigmoidalen Zugang eine Osteotomie des IAC nötig ist, da allein durch eine Analyse der präoperativen Bildgebung und das intraoperative Nervenmonitoring eine extrameatale Identifikation der Nervenstrukturen im Bereich des Hirnstamms nicht sicher möglich ist. Nachfolgend skizzieren wir daher unsere Ergebnisse der interdisziplinären intraoperativen Exploration des IAC mit Darstellung der Nervenverläufe bis zum Fundus für eine sichere morphologisch-anatomische Identifizierung des Nc beim retrosigmoidalen Zugangsweg bei Patienten mit einem VS im Tumorstadium Koos II–IV als potenzielle Kandidaten für eine Hörrehabilitation mit einem CI.

## Patienten und Methode

Zur Überprüfung unserer Ergebnisqualität (Studien-Nr. 2019-597) haben wir retrospektiv alle interdisziplinär retrosigmoidal operierten Patienten mit einem VS im Tumorstadium Koos II–IV mit einer reintonaudiometrisch mittelgradigen Schwerhörigkeit (gemittelter Hörverlust von mehr als 40 dB bei 0,5; 1; 2 und 4 kHz) bis an Taubheit grenzenden Schwerhörigkeit (elektroakustisch keine messbar reaktive Hörbahn im Sinne eines vorhandenen Residualhörvermögens auf der Tumorseite bei präoperativ fehlender objektivierbarer Welle V in der ABR bei einem Klickreiz zwischen 1 und 4 KHz und einem Stimulus bis 80 dB) [1] in der Zeit zwischen 2012 und 2020 ausgewertet. Ausgenommen wurden NFII-Patienten, Patienten mit einem VS im Tumorstadium Koos I (in diesem Tumorstadium lässt sich der Nc allgemein leicht identifizieren), Patienten mit einem VS im Tumorstadium Koos II oder III mit einer Normakusis (in diesen Fällen lässt sich der Nc intraoperativ ebenso mithilfe der ABR leicht und sicher extrameatal identifizieren), Patienten mit einer präoperativen Fazialisparese, Patienten mit einem Tumorrezidiv oder -residuum nach Voroperation oder einzeitiger/fraktionierter Strahlentherapie, Patienten mit Meningeomen oder anderen Tumorentitäten im Bereich des Kleinhirnbrückenwinkels (KHBW) sowie Patienten mit einem translabyrinthär, subtemporal oder retrolabyrinthär entfernten VS. Nach eingehender Beratung des Patienten über alle gängigen Therapieoptionen, „wait and scan“, stereotaktische Strahlentherapie durch γ‑Knife oder LINAC-Behandlung einzeitig oder fraktioniert oder über eine mikrochirurgische Resektion, erfolgte für das chirurgische Vorgehen immer eine präoperative Auswertung der radiologischen Datensätze (HCT Felsenbein und 3‑T- oder 3,5-T-MRT Schädel). Beurteilt wurde anhand der radiologischen Datensätze präoperativ die Position und Länge des IAC sowie, wenn möglich, die topografische Lage des Nf und Nc unter Berücksichtigung möglicher pneumatisierter Mastoidzellen. Ein anfänglich von uns durchgeführtes Cybertracking [7] des Nf auf Basis einer Brainlab-Plattform (Brainlab AG, München, Deutschland) als Navigationssystem für den präoperativ anzunehmenden Verlauf des Nf am Tumor hatte sich für uns nicht bewährt. Zum einen war bei einem kompletten Ausfüllen des IAC durch das VS der Nf in der CISS-3-D-MRT in der schräg-koronaren Projektion nicht mehr sicher identifizierbar, zum anderen ergab sich auch durch die notwendige chirurgisch-ablative Manipulation am Tumor eine ungewollte Verlagerung des Nf und Nc, allein schon durch die Tumorvolumenreduktion, sowie ein weiterer Zeitverlust für die Einrichtung des Navigationssystems beim präoperativen Setup, welches ohnehin schon durch die Patientenlagerung in der Park-Bench-Position mit Anlegen des Neuromonitoring viel Zeit in Anspruch nahm.

**Figure 1:**
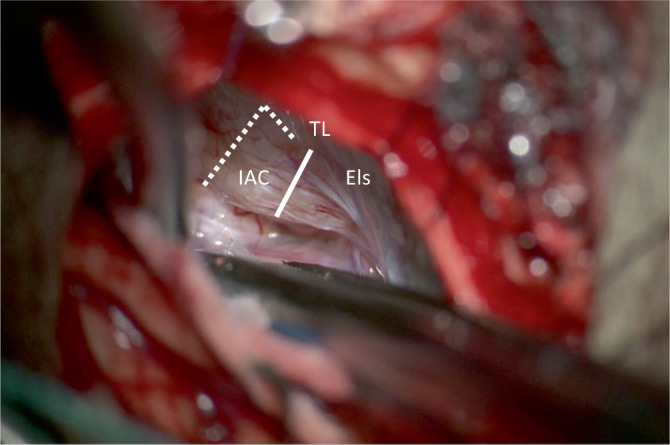

**Figure 2:**
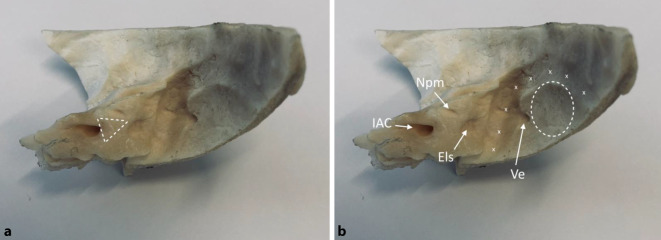

Intraoperativ führten wir für eine sichere Identifizierung des Nf und des Nc standardmäßig bei allen retrosigmoidal operierten Patienten nach Exploration des KHBW mit Eröffnung der hinteren Zisterne zunächst eine Fensterung der arachnoidalen Pseudokapsel des VS durch und reduzierten das Tumorvolumen durch Aushöhlung des Tumors unter Schonung des in der Pseudokapsel verlaufenden neurovaskulären Bündels, bei den Koos-III- und -IV-Tumoren dann auch mit dem CUSA (Cavitron Ultrasonic Surgical Aspirator). Anschließend erfolgte die ablative Osteotomie des IAC mit Aufsicht (mikroskopisch) oder endoskopisch assistierter Sicht bis zur Crista transversa (Abb. 1). Zur Durchführung der ablativen Osteotomie des IAC (Abb. 2a,b und 3) dienten als anatomische Landmarken zum einen der von einem Duramantel eingekleidete obere Eingang zum IAC, der Els („endolymphatic sac“) sowie der Austrittspunkt des Npm (Nervus petrosus major) als laterale Begrenzung. Durch die sichere Identifizierung des Els kann somit aus HNOKHC-Sicht unter Berücksichtigung des präoperativen HCT des Felsenbeins eine unbeabsichtigte Eröffnung des duralen Weichteilschlauchs verhindert und so der Nf und Nc sicher geschützt werden (Abb. 4). Von der Tübinger Arbeitsgruppe wurde in diesem Zusammenhang als anatomische Landmarke die sog. Tübinger Linie beschrieben ([4]; Abb. 1). Der Einsatz eines möglichen Hirnspatels erfolgt dabei immer nur temporär, um für kurze Zeit eine bessere Aufsicht auf den Hirnstamm zu erreichen. Während der gesamten operativen Maßnahmen wurde zur Prävention möglicher vaskulärer Spasmen insbesondere der Arteria labyrinthi kontinuierlich mit einer Nimodipin-Spüllösung gespült [30].

**Figure 3:**
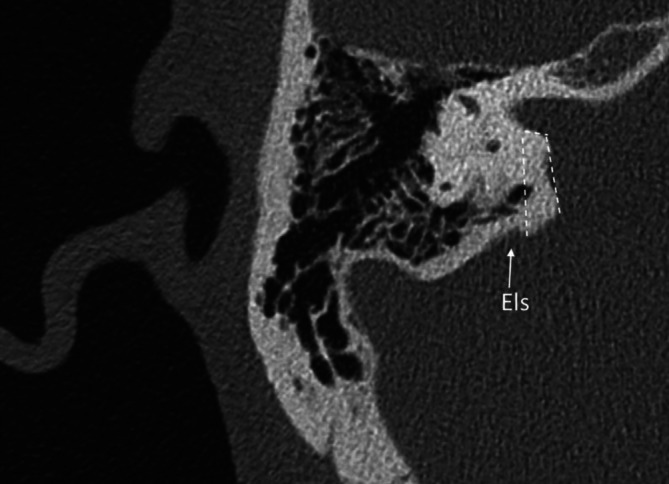

**Figure 4:**
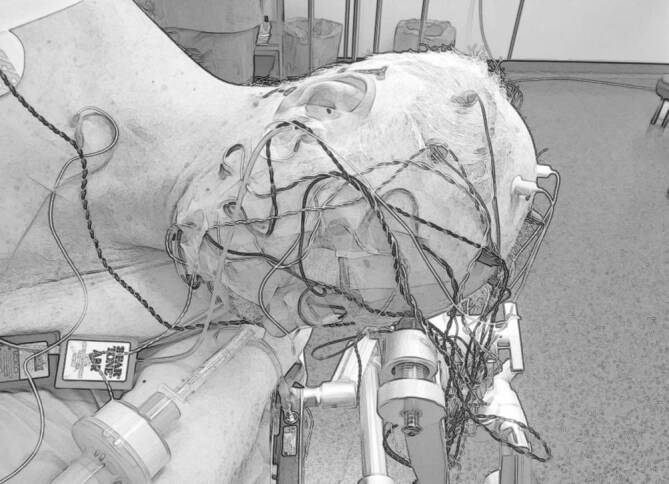

**Figure 5:**
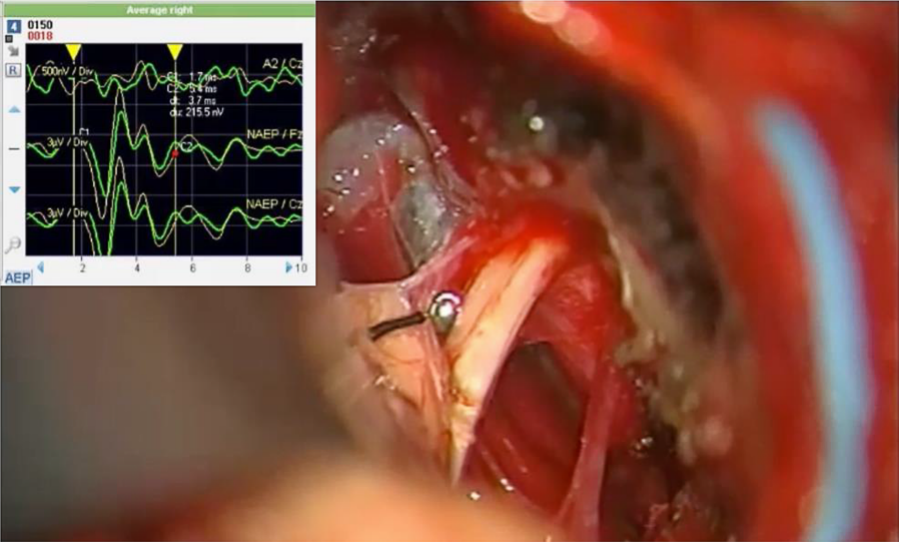

Alle Operationen erfolgten dabei unter einem kontinuierlichen Neuromonitoring des Nf, teilweise des Nc, sofern vor Beginn der Operation die Welle V in der ABR („auditory brainstem response“) bei wiederholter akustischer Stimulation zu mitteln war. Das Neuromonitoring des Nf erfolgte dabei mit einem NIM-Response‑3.0®-Gerät der Fa. Medtronic mit kontinuierlicher Kontrolle der EMG-Aktivität sowohl akustisch als auch visuell. Die funktionelle Überwachung des Nc, der kaudalen Hirnnerven sowie der motorischen und sensiblen Nervenbahnen erfolgte mittels eines Isis-Xpress-Mehrkanal-Neuromonitoring-Systems der Fa. inomed (Abb. 4). Für die funktionelle Überwachung des Nc mittels transtympanaler akustischer Stimulation und einer neuroelektrisch-enzephalographischen Ableitung wurde sowohl eine sog. Nahfeld- als auch Fernfeldmessung durchgeführt. Für die Nahfeldmessung wurde ein spezielle Kugelelektrode (Fa. inomed) intraoperativ auf den Nc gelegt (Abb. 5), die Fernfeldmessung erfolgte im messtechnischen Aufbau wie bei einer klassischen BERA („brainstem evoked response audiometry“) bzw. ABR („auditory brainstem response“). Im Gegensatz zur klassischen intraoperativen BERA als Fernfeldmessung wird bei der Nahfeldmessung mit der intraoperativ auf den Nc zu positionierenden Kugelableitelektrode die Welle 2 und 3 als Antwortpotenzial vom zweiten und dritten Neuron der Hörbahn arithmetisch gemittelt (CNAP, „cochlear nerve action potential“) [29].

Die intraoperativ identifizierte topografische Lage des Nf und Nc im IAC sowie im extrameatalen Verlauf wurde für jeden Patienten erfasst. Hierbei erfolgte eine systematische Auswertung der Lagebeziehung der beiden Nerven zueinander sowie dem vom Nv ausgehenden Tumor. Ziel war es, hierdurch analog zu den für das Koos Stadium II beschriebenen Subklassifikationen (Koos-Subtyp 1 A–C, 2 A/B, 3 A–C) übergreifend wiederkehrende Verlaufsmuster zu identifizieren und systematisch zu skizzieren.

Der intraoperative Verlauf des Nf und des Nc zum VS wurde, soweit möglich, im Vergleich mit der präoperativ laut CISS-3-D-MRT in der schräg koronaren Projektion vorhergesagten Lage der Nerven analysiert.

## Ergebnisse

Berücksichtigt werden konnten in der retrospektiven Analyse insgesamt 86 Patienten mit einem VS im Tumorstadium Koos II–IV, die auf der Tumorseite präoperativ sämtlich bereits reintonaudiometrisch mittelgradig schwerhörig bis taub waren. Bei allen Patienten wurde das VS mikrochirurgisch über einen retrosigmoidalen Zugangsweg entfernt (22 Patienten im Tumorstadium Koos II, 29 Patienten mit Tumorstadium Koos III und 35 Patienten im Tumorstadium Koos IV; Tab. 1). Hiervon zeigten insgesamt 11 Patienten im Tumorstadium Koos II präoperativ ein „verwertbares“ Restgehör, was einem gemittelten Hörvermögen von < 80 dB bei 0,5; 1; 2 und 4 kHz (nach WHO-Klassifikation Grad 2–3) entsprach und somit noch für eine potenzielle Hörgeräteversorgung infrage kam. Die anderen 75 Patienten zeigten bereits präoperativ ein fehlendes Antwortsignal (Welle V) in der ABR im Sinne eines Verlusts des sozialen Gehörs bis zur kompletten Ertaubung (Tab. 2). Aufgrund des retrospektiven Designs der Studie lag nur bei einem Teil der Patienten ein prä- und/oder postoperatives Sprachaudiogramm vor, sodass eine durchgehende Einteilung der Hörklassen nach AAO-HNS oder Gardner-Robertson nicht möglich war. Präoperativ lag bei 30 Patienten ein Freiburger Einsilbertest vor. Hierbei wurde allerdings nicht immer, wie für die Anwendung der AAO-HNS-Klassifikation im deutschsprachigen Testmaterial gefordert [25], das maximale Einsilberverstehen bestimmt, sondern häufig nur das Einsilberverstehen bei 65 dB. Unter Berücksichtigung dieser Einschränkungen und Verwendung des Mittelwerts von Reintonhörschwellen („pure-tone average“, PTA) bei 0,5; 1; 2 und 3 kHz lag bei 4 Patienten eine Hörklasse B nach der AAO-HNS-Klassifikation vor. Richtet man sich nach der von Wade und House beschriebenen 50/50-Regel, beschreibt dies ein im Alltag funktionales und gut versorgbares Hörvermögen [25].

**Table 1:** 

Tumorgröße nach Koos				
	Koos I	Koos II	Koos III	Koos IV
Patienten	–	22	29	35

**Table 2:** 

**Hörvermögen nach WHO-Klassifikation präoperativ**					
*–*	*WHO 0*	*WHO I*	*WHO II*	*WHO III*	*WHO IV*
Patienten	–	–	6	5	75
**Hörvermögen nach WHO-Klassifikation postoperativ**					
*–*	*WHO 0*	*WHO I*	*WHO II*	*WHO III*	*WHO IV*
Patienten	–	–	3	1	82

Präoperativ lag bei allen Patienten eine radiologische Bildanalyse des IAC in einer hochauflösenden (High-Resolution)HR-MRT in der 3‑D-CISS-Sequenz (T2-FSE) in axialer oder schräg sagittaler Projektion sowie ein hochauflösendes CT (HCT) des Felsenbeins vor. Anhand der radiologischen Datensätze konnten präoperativ die Position und Länge des IAC beurteilt werden und in einigen Fällen bereits Hinweise auf die topografische Lage des Nf und Nc gewonnen werden. Allerdings ließ sich in der 3‑D-CISS-T2-FSE radiologisch das Nerven-Gefäß-Bündel präoperativ häufig nicht sicher identifizieren, vor allem dann, wenn der IAC bis hin zur Crista transversa durch das VS komplett ausgefüllt war.

**Figure 6:**
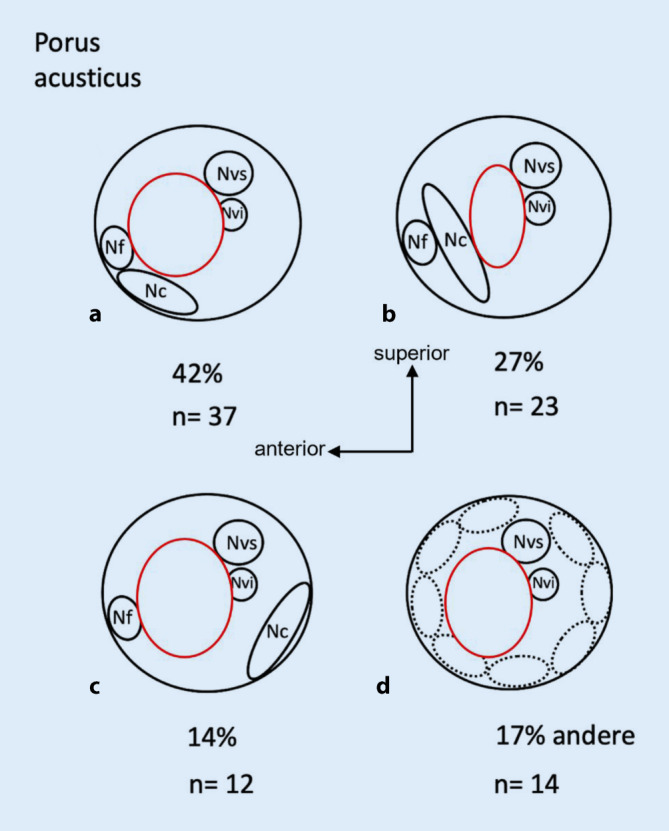

**Figure 7:**
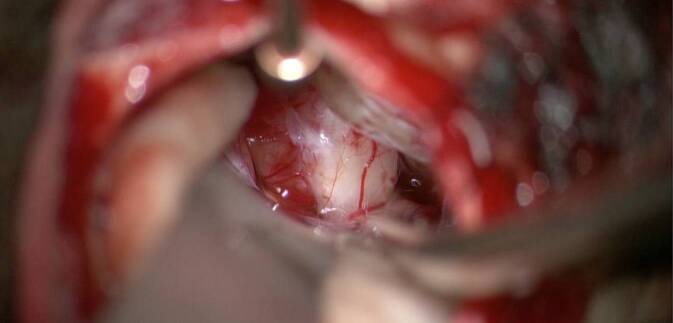

Intraoperativ erfolgte bei allen Patienten nach Schaffen des retrosigmoidalen Zugangswegs und Exploration des KHBW durch die NC eine Osteotomie des IAC durch die HNOKHC. Hierdurch ließ sich der NC mit seinem Austritt aus dem Fundus morphologisch-anatomisch sicher identifizieren. Auch innerhalb des IAC ließ sich dann der Nc auch ohne Antwortsignale in der ABR sowohl anhand seines Verlaufs als auch seines Verlaufsmusters korrespondierend zum Nf mit hoher Sicherheit identifizieren. Bei 72 (83 %) der 86 nachuntersuchten Patienten konnte somit der NC sicher identifiziert und im weiteren Verlauf der Op. die neuronale Struktur erhalten werden. Es zeigte sich, dass der Nc in der überwiegenden Zahl der Fälle einem bestimmten Verlaufsmuster zum Nf (Abb. 6) in Übereinstimmung mit der Literatur folgte [12, 14, 17]. Unabhängig vom Tumorstadium lagen bei 37 Patienten (42 %) der Nf und Nc am Porus acusticus anterokaudal nebeneinander. Bei 23 Patienten (27 %) kamen der Nf und NC ebenfalls anterokaudal zur Darstellung, allerdings nicht neben-, sondern hintereinander. Die beschriebene topografische Lage entsprach also dem von Koos für das Tumorstadium II beschriebenen Subtyp 1 B. Allerdings bildete die Koos-Subtyp-Einteilung hierbei nicht immer die absolute Realität ab, weil beispielsweise durch das verdrängende Tumorwachstum im IAC der Nf und Nc so stark aneinandergepresst wurden, dass sie fast miteinander verschmolzen (Abb. 7).

Bei 12 Patienten (14 %) lagen der Nf und Nc getrennt voneinander, der Nf kam hierbei anterokaudal, der Nc posterokaudal zu liegen. Dies entspräche im Tumorstadium Koos II dem Subtyp 2 A. Bei insgesamt 14 Patienten (17 %) ließ sich der Nc nicht sicher identifizieren.

Durch den somit insgesamt in der deutlichen Mehrheit vorhandenen unmittelbaren topografischen Kontakt zwischen Nf und Nc (Abb. 6) ließ sich der Nc neben dem Nf intraoperativ gut im IAC identifizieren.

Außerhalb vom IAC zum Hirnstamm hin war dies deutlich erschwert. Hier war eine Identifikation des Nc nur durch das Verfolgen des Nc aus dem IAC heraus in Richtung Hirnstamm oder mithilfe der Kugelelektrode, wenn als Nahfeldmessung der ABR diese ableitbar waren, möglich (Abb. 5). Insbesondere in den Fällen eines divergierenden Verlaufs des Nf und Nc (analog zum Koos-Subtyp 2 A oder B) sowie in den Tumorstadien III–IV war die Identifizierung ohne Verlaufsverfolgung vom Fundus herkommend oder ohne ABR des Nc erheblich erschwert, weil der Nc in diesen Fällen meistens mit der Tumorkapsel verbunden, ausgewalzt, aufgespreizt bzw. verschmolzen war. Diese Arten von VS waren charakterisiert durch eine hohe Adhärenz zwischen Tumorgewebe und der subarachnoidalen Tumorpseudokapsel, sodass häufig ein Erhalt des neuronalen Gewebes des Nc nur mit einem rasenförmigen Belassen von Tumorgewebe möglich war. Zudem war der Einsatz des CNAP für den Nc in solchen Situationen schwierig, weil sich die Ableitelektrode bei den Koos-III- und -IV-Tumoren tumorvolumenbedingt nicht am Abgang aus dem Hirnstamm platzieren ließ. In Fällen von zystischen oder zystisch-hämorrhagischen VS musste ein Teil der Tumorpseudokapsel zum Erhalt der neuronalen Strukturen des IAC immer erhalten werden.

Bezüglich des Funktionserhalts des Nc zeigte sich, dass in unserem Messaufbau die Welle V nicht immer prädiktiv für einen Hörerhalt bei den Koos-II-Tumoren war: Intraoperativ flachte die Welle V nicht selten ab, baute sich nach einer kurzen manipulativen Pause von wenigen Minuten dann nach entsprechender Mittelung wieder auf, ähnlich die Wellen II und III nach Stimulation mit der Kugelelektrode für die Nahfeldmessung, jedoch deutlich schneller. Trotz nachweislich vorhandener Welle V waren dann dennoch 4 von 11 Patienten mit einem noch „verwertbaren“ Gehör (WHO-Grad 2–3) mit einem Koos-II-VS auf der operierten Seite postoperativ reintonaudiometrisch taub (negativer prädiktiver Wert: 0,6; Tab. 2). In dem eingeschränkten Patientenkollektiv, welches nach AAO-HNS klassifiziert werden konnte, konnte nur bei 1 von 4 Patienten mit initial funktionalem Hörvermögen (Klasse B nach AAO-HNS) das Hören erhalten werden. Insofern decken sich unsere Ergebnisse bei Patienten mit einem Koos-II-VS und einem noch verwertbaren sozialen Gehör mit den Literaturergebnissen [19]. Limitierend für den Hörerhalt bei den Koos-II-VS war immer die Notwendigkeit einer möglichen Teilablation der Tumorpseudokapsel als Träger der neurovaskulären Strukturen des IAC, eine chirurgische Manipulation unmittelbar an der Arteria labyrinthi sowie eine notwendige Tumorablation angrenzend zur Crista transversa hin.

Bei 72 der 78 nachuntersuchten Patienten konnte schlussendlich durch die intraoperative Exploration des IAC der Nc identifiziert und im weiteren Verlauf der Op. die neuronale Struktur des Nc erhalten werden, sodass sich diese Patienten als potenzielle Kandidaten für ein CI zur Hörrehabilitation eigneten [13]. Die Funktion des Nervus facialis konnte in den meisten Fällen erhalten werden: Nach 12 Monaten zeigten noch 5 Patienten eine Fazialisfunktion im Stadium House-Brackman (HB) II–III, alle anderen 81 Patienten hatten keine Funktionseinschränkungen (HB I; Tab. 3).

**Table 3:** 

Fazialisfunktion nach House-Brackmann						
	HB I	HB II	HB III	HB IV	HB V	HB VI
Patienten	81	1	4	0	0	0

## Diskussion

Der retrosigmoidale Zugangsweg wurde erstmals von dem deutschen Anatomen und Chirurgen Fedor Krause 1903 beschrieben [15]. Der retrosigmoidale Zugangsweg als hörerhaltende Operationsmethode bei VS ersetzt in unserem interdisziplinären Schädelbasiszentrum immer mehr den subtemporalen und den deutlicher seltener anwendbaren retrolabyrinthären bzw. anterosigmoidalen Zugangsweg. Aus Sicht der HNOKHC wird dieser Zugangsweg etwas vernachlässigt, weil er immer eine entsprechend enge Abstimmung mit dem Nachbarfach NC erfordert [16]. Einfacher ist die Zusammenarbeit in einem von der Gesellschaft für Schädelbasischirurgie e. V. (GSB) zertifizierten Schädelbasiszentrum mit regelmäßigen wöchentlich dokumentierten interdisziplinären Fallkonferenzen. So führen wir nicht nur die Fallkonferenzen, sondern eben auch die schädelbasischirurgischen Interventionen regelmäßig gemeinsam interdisziplinär durch. Für die NC ist der retrosigmoidale Zugangsweg das „work horse“ für die hintere Schädelgrube und ist weitestgehend standardisiert [17, 34, 35]. Zugangsspezifische Komplikationen sind Verletzungen der Vena emissaria unmittelbar am Abgang aus dem Sinus sigmoideus oder des Sinus sigmoideus selbst mit entsprechendem Blutvolumenverlust, eine verzögerte Eröffnung der hinteren Subarachnoidalzisterne mit möglicher nachfolgender Schwellung des Zerebellums sowie eine ungewollte mechanische Irritation der kaudalen Hirnnerven. Der große Vorteil gegenüber dem subtemporalen und retrolabyrinthären Zugangsweg ist die sichere Kontrolle der AICA (Arteria cerebelli anterior inferior) und von deren Abgängen sowie der Subarachnoidalvenen sowie eine bessere Übersicht im Bereich des Hirnstamms bei Koos-III- und -IV-Tumoren. Die retrosigmoidale Eröffnung des IAC ohne Eröffnung des PSC zum Hörerhalt ist andererseits eine chirurgische Domäne der HNOKHC [6]. Gerade durch die chirurgische Kompetenz der HNOKHC lassen sich selbst ausschließlich intrameatal gelegene Koos-I-VS je nach anatomischer Position des PSC ohne Weiteres hörerhaltend alternativ zum subtemporalen Zugangsweg chirurgisch entfernen [3, 23, 27]. Durch die Möglichkeit einer erfolgreichen Hörrehabilitation mithilfe eines CI auch bei tumorbedingt bereits präoperativ ertaubten Patienten ergibt sich zukünftig auch die Notwendigkeit eines sicheren Kontinuitätserhalts des Nc. Dazu ist eine sichere intraoperative Identifizierung desselben notwendig, auch wenn in der intraoperativen ABR methodenbedingt der Nc bereits elektrophysiologisch inaktiv imponiert. Alternativ wäre nur eine eABR denkbar [36], die dann aber einen 2‑Höhlen-Eingriff erfordern würde und bereits präoperativ den Patientenwunsch nach einer Hörrehabilitation voraussetzt.

Für eine möglichst sichere anatomische Identifikation des Nc erfolgt eine retrosigmoidale, explorativ-ablative Osteotomie in dem Dreieck zwischen Npm und Els mit der Crista transversa des IAC als Scheitelpunkt (Abb. 2a,b). Die genaue geometrische Position des PSC zum Npm und Els muss immer aufs Neue individuell mithilfe des HRCT des Felsenbeins bestimmt werden. Die Crista transversa des IAC sollte nach Möglichkeit nur bis zu deren freien Aufsicht freigelegt werden, da der Nc beim Eintritt zur Lamina spiralis in Richtung Cochlea ohne die schützende Dura völlig frei liegt und hier leicht bei bereits kleinsten chirurgischen Manipulationen einreißt. Im Fall von der Crista transversa unmittelbar anliegenden Tumormassen eines VS belassen wir daher Tumorreste zum Hörerhalt, zumal die neuere Literatur gezeigt hat, dass durch die Kappung der nutritiven neoproliferierten Tumorgefäße das verbleibende Resttumorgewebe in dieser Lokalisation degenerieren und narbig obliterieren kann [8, 12, 32]. Bei einem vom IAC ausgehenden intracochleären oder intralabyrinthären Tumorwachstum ist sonst alternativ der translabyrinthäre Zugangsweg zu bevorzugen mit Versuch eines Erhalts des Nc und der Cochlea als Elektrodenlager für eine spätere Cochleaimplantation. Eine endoskopisch transmeatal, transcochleär geführte, mikrochirurgische Resektion eines VS würde mit einer Sakrifizierung der Cochlea als Elektrodenlager einhergehen und somit für den betroffenen Patienten die Möglichkeit einer späteren Hörrehabilitation mit einem CI gänzlich ausschließen.

Der Verlauf des Nf und des Nc innerhalb des Gefäßnervenbündels des IAC ändert sich zwischen dem Ausgang aus dem Hirnstamm bis hin zum Eingang zur Cochlea [11]. Die beiden Vestibularnerven Nvi und Nvs verlaufen nach Verlassen des Hirnstamms zunächst zusammen als Nv und trennen sich dann am medialen Eingang zum IAC in den Nvi und Nvs. Der Verlauf des Nc dreht dabei von posterior inferior nach anterior inferior, kann aber auch je nach Größe und Beschaffenheit des VS (kompakt homogen, zystisch oder hämorrhagisch heterogen) hiervon abweichen. Erste Informationen über den intraoperativ zu erwartenden Verlauf des Nf und Nc innerhalb des IAC erhält man möglicherweise in der HR-MRT in der CISS-Sequenz (T2-FSE) in axialer oder schräg sagittaler Projektion, wenn das VS den IAC noch nicht komplett ausgefüllt hat. Bei der Beratung des Patienten über die Möglichkeiten des Hörerhalts bzw. Erhalt des Nc in Abwägung der alternativ zur Verfügung stehenden Behandlungsverfahren ist immer die audiologische Kompetenz des kontralateralen Ohrs mit in Betracht zu ziehen. Im Fall eines gewünschten mikrochirurgischen Vorgehens kann dann eine Komplettentfernung des VS als gutartiger Tumor kontraproduktiv sein, weil, wie bei unseren ausgewerteten Fällen zu sehen war, sich trotz Kontinuitätserhalt und nachweisbarer Welle V dennoch ein Funktionsverlust des Nc ergeben kann. Daher kann es in solchen Fällen für den Erhalt der neuronalen Funktionsintegrität des Nc sinnvoll sein, Teile des bekanntermaßen als gutartig einzustufenden VS in diesem Bereich „rasenartig“ stehen zu lassen. Der dabei zu fordernde Kontinuitätserhalt des Nc bekommt durch die guten Ergebnisse bei SSD(Single-Side-Deafness)-Patienten mit einem CI zunehmend Bedeutung. War noch Anfang der 1970er- und 1980er-Jahre der Funktionserhalt des Nf maßgebend, so ist heute dessen Funktionserhalt nicht zuletzt durch das obligate Neuromonitoring selbstverständlich, und der Fokus gilt heutzutage durch die Möglichkeit einer Hörrehabilitation mit einem CI dem Funktions- und Kontinuitätserhalt des Nc [2, 13].

Die Ursprungsmatrix des VS ob vom Nvi oder Nvs ausgehend, gemessen am präoperativen KIT (Kopf-Impuls-Test) [24] spielte für die sichere Identifizierung des Nc und Nf in unserer Studie eine untergeordnete Rolle. Vielmehr fanden wir in über 2/3 der Fälle ein bestimmtes immer wiederkehrendes Verlaufsmuster des Nc korrespondierend zum Nf, der sich als motorischer Nerv elektroneurographisch immer sicher identifizieren ließ (Abb. 6). Anhand dieser immer wiederkehrenden Verlaufsmuster beider Nerven ließ sich bereits anhand des elektroneurographisch immer zu identifizierenden Nf der Nc ebenso lokalisieren und teilweise auch bei nachweisbaren Antwortpotenzialen mithilfe der Kugelelektrode in der Nahfeldmessung verifizieren. Somit kann man heutzutage intraoperativ den Kontinuitätserhalt des Nc in den meisten Fällen sicherstellen, auch wenn damit noch nicht der Funktionserhalt gewahrt ist. Für den Funktionserhalt des Nc scheinen neben einer möglichen Minderperfusion im Bereich der Arteria labyrinthi noch andere bislang unbekannte Faktoren von Bedeutung zu sein. Der Kontinuitäts- und Funktionserhalt des Nc ermöglicht für den Patienten eine Hörrehabilitation mit einem CI. Dabei ist jedoch zu berücksichtigen, dass es, ähnlich wie beim translabyrinthären Zugangsweg, auch beim retrosigmoidalen Zugangsweg nachfolgend zu einer narbigen Obliteration der Cochlea kommen kann [9]. Daher kann man simultan oder zeitnah konsekutiv einen Platzhalter für die Elektrode in die Cochlea einbringen, vor allem dann, wenn sich in der postoperativen MRT-Untersuchung in den T2-CISS-Sequenzen erste Hinweise auf eine beginnende Vernarbungstendenz in Form eines fehlenden Flüssigkeitssignals in der 1. oder 2. Windung der Cochlea zeigen [2].

Für den Funktionserhalt des Nc hat sich im Zusammenhang mit den ABR-Messungen und in Übereinstimmung mit der Literatur ergeben, dass intraoperativ wiederholt Pausen bis zur Erholung der Wellen 2 bis 5 günstig sein können [8, 12, 32]. Welche physiologischen metabolischen Prozesse hierfür verantwortlich sind, ist jedoch bis heute immer noch nicht im Einzelnen bekannt, sodass auch nicht klar ist, in welchen Situationen genau solche Pausen angezeigt sind und wie lange sie für den Funktionserhalt des Nc eingehalten werden sollten. In der Literatur ist das Risiko für eine postoperative Ertaubung nach solchen temporären Veränderungen/Verlust der Welle V mit 54–70 % angegeben [21]. Dies kann auch nach kompletter Erholung der Welle V am Ende der Messungen der Fall sein, was eine mögliche Erklärung für die von uns beschriebene Diskrepanz zwischen intraoperativ erhaltener Welle V und dennoch eingetretener postoperativer Ertaubung sein kann. Eine weitere mögliche Erklärung hierfür wäre ein verzögertes Eintreten des Hörverlusts, welcher nicht unmittelbar durch die chirurgische Manipulation, sondern z. B. durch eine verzögert eintretende Ischämie der Cochlea (z. B. durch Vasospasmen), Fibrose oder Labyrinthitis bedingt sein kann [33]. Eine gewisse Unsicherheit in der Vorhersagekraft der ABR-Messung ist ggf. auch durch die Tatsache bedingt, dass es sich um eine subjektive Auswertung handelt, die z. B. von der Erfahrung der auswertenden Person abhängig sein kann und teilweise durch Artefaktüberlagerungen erschwert sein kann [26].

Die Ergebnisse zum Hörerhalt bei den Koos-II-VS mit einem präoperativ noch „verwertbaren“ Hörvermögen (7 von 11 Patienten postoperativ mit einem weiterhin noch verwertbaren Hörvermögen) bestätigen die Literaturdaten, dass bei dieser Tumorgröße und einem guten audiologischen Ausgangsbefund die VS am besten mikrochirurgisch zu entfernen sind [8, 12, 32]. Allerdings zeigt der quantitative Zusammenhang zwischen Veränderungen in der ABR und dem Grad des postoperativen Hörvermögens eine erhebliche Varianz, und es sind sowohl falsch-positive als auch wie bei uns beschrieben falsch-negative Ergebnisse möglich [26]. Somit scheint weder der anatomische Erhalt der Nervenstrukturen noch der intraoperative Nachweis der Welle V eine sichere Aussage über den Funktionserhalt des Nc treffen zu können. Da dieser aber ausschlaggebend für das spätere Rehabilitationsergebnis der Cochleaimplantatversorgung ist, sollte der Patient über eine entsprechende Unsicherheit in der Erwartungshaltung aufgeklärt werden. Als Alternative ist bei diesen Patienten eine sog. Wait-&-Scan-Strategie zu diskutieren. Bei zu langem Zuwarten kann diese den Nachteil haben, dass es zu einer nicht mehr umkehrbaren tumorbedingten neuronalen Schädigung am Nc kommt und somit der optimale Zeitpunkt einer Operation verpasst wird. Als weitere Alternative muss eine frühe Strahlentherapie für diese Patienten diskutiert werden. Im Gegensatz zu den nicht selten auftretenden Hörstürzen mit anschließender Erholung des Hörvermögens im Zusammenhang mit einem VS wird ein langsam fortschreitender Hörverlust vom Betroffenen zunächst nicht als störend empfunden. Dies kann dazu führen, dass solche Tumoren nicht selten erst im Tumorstadium Koos III oder IV mit einer deutlich ungünstigeren Prognose für den Hörerhalt erkannt werden.

## Fazit

Der Nervus cochlearis verläuft in über 2/3 der Fälle korrespondierend zum Nervus facialis nach einem bestimmten Verlaufsmuster im Bereich des IAC und lässt sich somit ausgehend vom Fundus gut identifizieren. Eine Axono- oder Neurotmesis des Nervus facialis ist heutzutage durch Einsatz des Neuromonitoring mit wenigen Ausnahmen sehr unwahrscheinlich geworden. Der Funktions-, zumindest aber der anatomische Kontinuitätserhalt des Nervus cochlearis sollte, wenn möglich, immer angestrebt werden, um eine eventuelle spätere Versorgung mit einem CI zu ermöglichen.
