# Erratum zu: Kontinuitätserhalt des Nervus cochlearis bei der retrosigmoidalen ablativen Osteotomie des inneren Gehörgangs bei fortgeschrittenen Vestibularisschwannomen

**DOI:** 10.1007/s00106-021-01138-6

**Published:** 2022-01-18

**Authors:** Katharina Schaumann, A. Albrecht, B. Turowski, C. Hoffmann, J. F. Cornelius, J. Schipper

**Affiliations:** 1 Universitätsklinik für Hals‑, Nasen- und Ohrenheilkunde und Poliklinik, Heinrich-Heine-Universität Düsseldorf, Moorenstr. 5, 40255 Düsseldorf, Deutschland; 2 Institut für diagnostische und interventionelle Radiologie, Heinrich-Heine-Universität Düsseldorf, Düsseldorf, Deutschland; 3 Universitätsklinik für Neurochirurgie, Heinrich-Heine-Universität Düsseldorf, Düsseldorf, Deutschland

**Erratum zu:**

**HNO 2021**

10.1007/s00106-021-01116-y

In diesem Artikel wurde der Name des Autors J. F. Cornelius fälschlicherweise als J. Cornelius angegeben.

The original article has been corrected.

